# Proteomic analysis of the extraembryonic tissues from cloned porcine fetus at day 35 of pregnancy

**DOI:** 10.1186/1756-0500-7-861

**Published:** 2014-11-29

**Authors:** Yeoung-Gyu Ko, Seongsoo Hwang, Sung Woo Kim, Hyun Kim, Hwan Hoo Seong, Jae-Hwan Kim, Yuno Song, Bo-Suck Yang, Young Min Song, Jae-Hyeon Cho

**Affiliations:** Animal Genetic Resources Station, National Institute of Animal Science, RDA, Namwon, 590-832 Korea; Institute of Agriculture and Life Science, College of Veterinary Medicine, Gyeongsang National University, Jiju Daero 501, Jinju, Gyeongsangnamdo, 660-701 Korea; Department of Animal Science & Biotechnology, Gyeongnam National University of Science and Technology, Jinju, 660-758 Korea

**Keywords:** Extraembryonic tissue, Placenta, SCNT, Apotosis, Gene expression

## Abstract

**Background:**

Somatic cell cloning by nuclear transfer (SCNT) in pig is clearly of great benefit for basic research and biomedical applications. Even though cloned offspring have been successfully produced in pig, SCNT is struggling with the low efficiency.

**Results:**

In the present study, we investigated differentially expressed proteins of the extraembryonic tissue from pig SCNT fetus compared to control (normal) fetus. We obtained the extraembryonic tissue from embryos at day 35 of pregnancy and examined the protein expression profiles using two-dimensional electrophoresis (2-D) and Western blotting. The extraembryonic tissue of fetus in control pregnancy was compared to the extraembryonic tissue of SCNT fetus, which showed an abnormally small size and shape as well as exhibited abnormal placental morphology compared to control fetus. A proteomic analysis showed that the expression of 33 proteins was significantly increased or decreased in the extraembryonic tissue of SCNT fetus compared to control fetus. The differentially expressed proteins in the extraembryonic tissue of SCNT fetus included ATP or lipid binding proteins, antioxidant proteins, translation elongation factors, and transcription factors. Western blotting analysis indicated that antioxidant enzymes and anti-apoptotic proteins were down-regulated; however, the expression levels of apoptotic proteins, Bax and Hsp27, were increased in the extraembryonic tissue of SCNT fetus. Moreover, immunohistochemical analysis also showed that the expression of the catalase or GPX genes was decreased in the extraembryonic tissue with SCNT fetus compared to those with control fetus. In addition, we observed a significant decrease in DNA methytransferase1 (Dnmt1) expression in SCNT extraembryonic tissue, and the expression levels of Dnmt3a and Dnmt3b were abnormally higher in SCNT fetus compared to control fetus. Moreover, a marked increase in the frequency of TUNEL-positive cells was observed in the extraembryonic tissue in SCNT fetus.

**Conclusion:**

These results demonstrated that pig SCNT fetus showed abnormal protein expression in the extraembryonic tissue, and extensive apoptosis occurred in the extraembryonic tissue of the SCNT fetus due to an increase in apoptotic protein expression or a decrease in antioxidant protein expression.

## Background

The success of somatic cell nuclear transfer (SCNT) in pigs is promising for a wide range of applications, such as genetically superior pig breed production, species resource preservation and xenotransplantation in humans [1, 2]. The birth of cloned pigs is achieved by the nuclear transfer of the nuclei of somatic cells [3, 4]. However, early embryonic mortality in SCNT embryos pregnant pigs is high during the first 30 days of pregnancy. The high rates of embryonic death of SCNT embryos are thought to be a result of inadequate nuclear reprogramming and extraembryonic tissue formation defects in the cloned embryo. Therefore, the majority of failed pregnancies appears to result from abnormal extraembryonic tissue development, such as reduced vascularization and enlarged placentomes [5–7].

In the genome of SCNT embryos, abnormal methylation patterns were detected in cloned embryos compared to embryos derived by in vitro fertilization [8]. Trophectoderm-localized methylation aberrancy was observed and is related to placental dysfunction closely observed in cloned animals [9]. The abnormal epigenetic reprogramming of trophoblasts in SCNT embryos suggests that the extraembryonic tissue of trophoblasts develops abnormally. Aberrant methylation in the trophectoderm of cloned blastocysts induces global gene dysregulation in extraembryonic tissues, and this type of gene dysregulation can potentially lead to the development of a dysfunctional placenta and have detrimental effects on fetal development [10, 11].

Apoptosis is an important process during animal development and reproduction. During pregnancy, apoptosis is physiologically important for normal placental growth and development [12]. Apoptosis is triggered by many different cellular stimuli, including oxidative stress, oxidative damage and cytotoxicity, which induce the activation of the caspase-cascade signaling system. Oxidative stress contributes to ROS formation in SCNT fetus, and ROS levels gradually increase during early pregnancy, resulting in apoptotic cell death in SCNT fetus [13]. Koo and colleagues demonstrated that oxidative stress is a major cause of apoptosis in SCNT extraembryonic tissue and that the low birth rate of cloned animals is due to abnormal apoptosis in the extraembryonic tissue during early pregnancy [14].

In the present study, we investigated differences in gene expression profiles of the extraembryonic tissue of SCNT and control fetus at early pregnancy (gestational day 35) using two-dimensional electrophoresis (2-D) analysis. In the proteomic analysis, 33 proteins were identified as being differentially regulated in the SCNT extraembryonic tissues. Among these proteins, 3 proteins were up-regulated, whereas the other 30 proteins were down-regulated. Proteomic analysis showed that the expression of apoptosis-related proteins, Bax and Hsp27 were expressed significantly higher in SCNT than that of the normal extraembryonic tissue, whereas the majority of the ATP binding proteins, antioxidant proteins, and metabolic-related proteins were down-regulated in SCNT extraembryonic tissues. The results of this study showed that the expression profile of the genes in extraembryonic tissue of SCNT fetus s had increased apoptosis-related proteins and decreased oxidative stress proteins, causing abnormal placenta and fetal development and increased embryonic loss during early pregnancy.

## Results

### Morphology of the fetus and extraembryonic tissue

**Figure 1:**
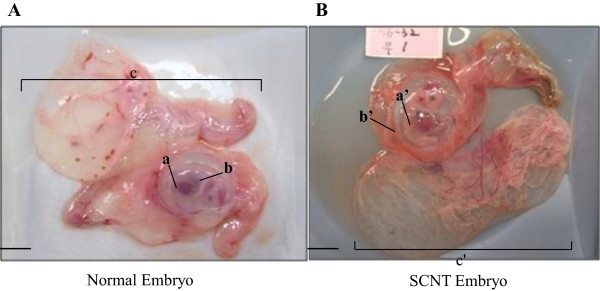
**Morphology of fetus and extraembryonic tissues in the control and SCNT-derived fetus from 35 day of pregnancy. (A)** Control fetus and extraembryonic tissue from 35 day of pregnancy. **(B)** The SCNT fetus and extraembryonic tissue from 35 day of pregnancy. a and a’, embryo; b and b’, amnionic sac; c and c’ extraembryonic tissue. The weight of fetus was measured after removal of all extraembryonic tissues including chorioallantois, amniotic and allantoic fluid, and umbilical cord. Bar, 10 mm.

A total of 1500 nuclear transferred embryos at the 1- to 2-cell stage were surgically transferred to the oviducts of 18 recipients. Eight recipients (8 pregnancy/18 recipient) were confirmed to be pregnant at day 35 after the transfer of the embryo. As shown in Table 1, the number of recovered SCNT-derived fetus (44.4% pregnancy) at gestational day 35 is significantly lower than the number of fetus recovered from the control (100% pregnancy) (Table 1). The control conceptuses (normal) were obtained from recipients that were impregnated by natural mating. The size and shape of fetus and extraembryonic tisssues were observed in the microscopic analysis. SCNT extraembryonic tissue from day 35 of pregnancy was smaller in size and more abnormal in appearance compared to the control extraembryonic tissue (Figure 1). Abnormal blood vessel development was observed in the extraembryonic tissue of SCNT fetus. Interestingly, the sizes of the allantoic and the amniotic sac of both SCNT fetus and control fetus were similar. The fetal weight of the SCNT groups (2.71 ± 0.4) was significantly smaller than the weight of the control groups (3.34 ± 0.26) (Table 1). The number of recovered fetuses from the control group (11.33 ± 1.33) was higher than the number recovered from the SCNT group (4.67 ± 2.33) (Table 1). The number of abnormal fetus with SCNT group (2.33 ± 0.33) was significantly higher than the control group (1 ± 0) (Table 1). Together, these results demonstrated that the extraembryonic tissue in the SCNT fetus showed an abnormally small size and shape, and displayed abnormal morphology of the extraembryonic tissue compared with control (natural mating).

**Table 1:** **Fetal body weight and the number of fetuses recovered at day 35 of pregnancy**

Animal group	Recipients	Pregnancy (%)	No. fetuses recovered		Body weight (g)
			Normal	Abnormal	
Control	10	100	11.33 ± 1.33	1 ± 0	3.34 ± 0.26
SCNT	18	44.4	4.67 ± 2.33	2.33 ± 0.33	2.71 ± 0.4

Control, natural mating; SCNT, SCNT fetus. The number mean of normally recovered fetuses from the one SCNT-recipient gilt was 4.67 ± 2.33. The ratios of control fetal weight were significantly higher than those of the SCNT fetus. Data were expressed as mean ± SD.

### 2-D gel analysis of the extraembryonic tissue from control and SCNT fetus

**Figure 2:**
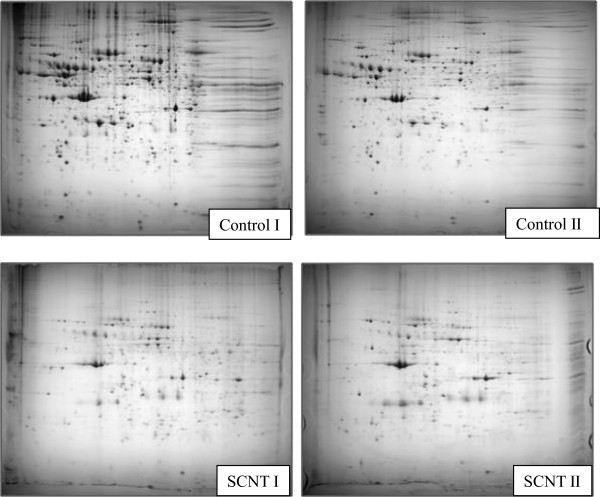
**Two-dimensional protein separation of the control and SCNT extraembyonic tissue as visualized by the silver staining.** Proteins were isolated from the control and SCNT extraembryonic tissue, and 1000 μg of total protein were loaded to the 2-D gel. The first dimension was 24-cm pH 4–10 nonlinear IPG, and the second dimension was 10 ~ 16% gradient gels (Control I and II, control extraembryonic tissues; SCNT I and II, SCNT extraembryonic tissues).

To examine protein expression differences between control and SCNT fetus, we performed proteomics analysis using the extraembryonic tissue of SCNT and control fetus on day 35 of pregnancy. Figure 2 shows the profile of extraembryonic proteins from SCNT and control fetus using 2-D with a nonlinear IPG gradient of pH 4–10 and 10 ~ 16% gradient gels in the second dimension. A representative 2-D image of proteins in the control and SCNT extraembryonic tissue is shown in Figure 2, and 33 spots were identified as shown in Tables 2 and 3. After protein identification with mass spectrophotometry and protein database search, 3 proteins were up-regulated (2.0- to 4.5-fold increases) and 30 proteins were down-regulated (2.0- to 5.5-fold decreases) in the SCNT extraembryonic tissue (Tables 2 and 3). Tables 2 and 3 summarizes the primary properties of the 33 proteins that were differentially regulated in SCNT and control fetus. The proteins are classified into six groups based on function: (1) lipid binding proteins (2/33, 6%), (2) antioxidant proteins (3/33, 9%), (3) translation elongation factors (3/33, 9%), (4) ATP binding proteins (6/33, 18%), (5) transcription factors (2/36, 6%), and (6) others (15/36, 51%). The majority of the ATP binding proteins, antioxidant proteins, and metabolic-related proteins were down-regulated, whereas the expression levels of peroxisome proliferative activated receptor gamma (PPARγ), Hsp27, and elongation factor 1-gamma (EF1-γ) were up-regulated in SCNT extraembryonic tissue.

**Table 2:** **Up-regulated proteins in the SCNT extraembryonic tissue**

Spot no.	Protein name	NCBI accession no.	Species	PMF (MS)			Experimental		Ideal	
				Matched peptides	Measured peptides	Sequence coverage (%)	Molecular mass (kDa)	pI	Molecular mass (kDa)	pI
2401	peroxisome proliferative activated receptor gamma	AAN15206.1	Ovis aries	8	16	23	54.95	6.2	46.63	4.60
5110	heat shock 27 kDa protein 1	NP_001007519.1	Sus scrofa	11	13	43	22.98	6.2	28.02	5.80
5118	Elongation factor 1-gamma (EF-1-gamma) (eEF-1B gamma)	spQ29387	Sus scrofa	7	8	18	49.95	6.2	13.90	5.72

After protein identification with mass spectrophotometry and protein database search, 3 proteins were up-regulated (2.0-to 4.5-fold increases) in the SCNT extraembryonic tissue.

**Table 3:** **Down-regulated proteins in the SCNT extraembryonic tissue**

Spot no.	Protein name	NCBI accession no.	Species	PMF (MS)			Experimental		Ideal	
				Matched peptides	Measured peptides	Sequence coverage (%)	Molecular mass (kDa)	pI	Molecular mass (kDa)	pI
2106	apolipoprotein A-I	AAA30992.1	Sus scrofa	24	24	61	30.31	5.4	26.42	4.67
1203	translational elongation factor 1 delta	NP_001009449.1	Ovis aries	9	9	27	30.97	5.4	38.83	4.43
1204	calpain, small subunit 1	NP_999483.1	Sus scrofa	8	14	21	28.16	5.0	29.96	4.45
1312	thioredoxin-like 1	NP_004777.1	Homo sapiens	13	14	48	32.63	4.8	39.48	4.57
1409	cytokeratin 19	NP_001015600.1	Bos taurus	14	24	33	43.87	4.9	47.00	4.52
1511	beta 5-tubulin	BAD08435.1	Sus scrofa	28	28	43	50.11	4.8	63.04	4.47
1601	gamma-aminobutyric acid (GABA) A receptor, alpha 2	1411303A	Bos taurus	10	13	23	51.46	9.3	67.05	4.32
1808	90-kDa heat shock protein alpha	NP_999138.1	Sus scrofa	26	28	38	85.11	4.9	112.70	4.53
1811	heat shock protein 90 beta	BAB20776.1	Equus caballus	28	42	35	82.29	5.0	112.11	4.58
2107	eukaryotic translation initiation factor 5A	NP_001003658.1	Bos taurus	8	12	42	17.04	5.1	13.78	4.69
2108	Peroxiredoxin-2 (Thioredoxin peroxidase 1) (Thioredoxin-dependent peroxide reductase 1) (Thiol-specific antioxidant protein) (TSA)	spP52552	Sus scrofa	6	6	34	13.82	4.7	23.67	4.72
2201	epsilon1-COP	BAA94967.1	Bos taurus	14	17	32	34.69	5.0	36.64	4.60
2203	cytochrome b	AAR10359.1	Phoca caspica	5	9	15	40.42	6.6	31.74	4.65
2204	Rho GDP dissociation inhibitor (GDI) beta	NP_786991.1	Bos taurus	5	6	30	22.83	5.1	29.40	4.67
2205	carbonic anhydrase III	NP_001008688.1	Sus scrofa	12	23	56	29.68	7.9	31.26	4.69
2206	pro alpha 1(I) collagen	CAC38832.1	Bos taurus	7	11	20	32.14	5.5	38.27	4.70
2301	tropomodulin 3 (ubiquitous)	NP_999459.1	Sus scrofa	13	15	41	39.74	5.0	45.66	4.67
2305	apolipoprotein E precursor	NP_999473.1	Sus scrofa	15	19	26	36.64	5.6	39.77	4.85
2610	Vimentin	spP02543	Sus scrofa	13	21	59	31.03	6.5	64.64	4.74
2611	Tubulin alpha-1A chain (Tubulin alpha-1 chain) (Alpha-tubulin 1)	spP02550	Sus scrofa	16	25	43	50.80	4.9	67.27	4.75
2709	6A3-5 protein	AAT75226.1	Sus scrofa	13	17	6	188.33	6.1	86.97	4.83
2815	valosin-containing protein	NP_999445.1	Sus scrofa	19	22	23	89.96	5.1	119.25	4.89
3206	apolipoprotein E precursor	NP_999473.1	Sus scrofa	23	25	44	36.64	5.6	38.43	5.00
3412	Eukaryotic initiation factor 4A-I (ATP-dependent RNA helicase eIF4A-1) (eIF4A-I) (eIF-4A-I)	spP29562	Oryctolagus cuniculus	29	31	59	45.50	5.3	50.39	5.11
3703	Structural maintenance of chromosomes protein 2 (Chromosome-associated protein E) (XCAP-E homolog) (FGF-inducible protein 16)	spQ8CG48	Mus musculus	13	14	15	134.77	8.7	98.12	4.94
3801	transglutaminase 2	NP_803473.1	Bos taurus	10	17	20	78.29	5.1	103.17	4.93
3805	valosin-containing protein	1303334A	Sus scrofa	34	38	43	89.97	5.1	118.87	4.95
5105	retinol-binding protein 4, plasma	spP27485	Sus scrofa	12	12	60	23.39	5.4	23.05	5.61
5117	stathmin-1	NP_001009582.1	Sus scrofa	5	5	29	17.28	5.8	13.97	5.52
6701	albumin	CAA30970.1	Sus scrofa	24	25	41	71.39	5.9	82.15	6.12

The 30 differentially down-regulated proteins (2.0-to 5.5-fold decreases) were identified according to protein identification with mass spectrophotometry and protein database search.

### Immunohistochemical analysis in control and SCNT extraembryonic tisssues

**Figure 3:**
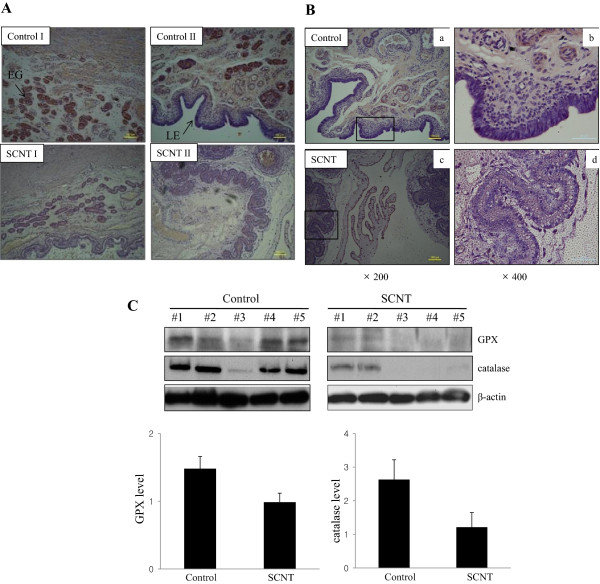
**Immunohistochemical analysis of antioxidant proteins in the control and SCNT extraembryonic tissue. (A)** Catalase expression in the control extraembryonic tissue was mainly localized on the luminal epithelium (LE) and endometrial glandular (EG) cells. Magnification: control and SCNT extraembryonic tissue (x200). Scale bars represent 100 μm. **(B)** Immunohistochemical analysis of GPX in the control and SCNT extraembryonic tissue. GPX was strongly expressed in the luminal epithelium (LE) of the control extraembryonic tissue. The insect in (a, x200) and (c, x200) showed (b, x400) and (d, x400), respectively. Scale bars represent 100 μm. **(C)** Western blot analysis of antioxidant enzyme proteins in the control and SCNT extraembryonic tissue. The expression of GPX and Catalase was down-regulated in the SCNT extraembryonic tissue.

To determine the effect of the abnormal development of extraembryonic tissue in SCNT fetus, we performed immunohistochemical analysis of control and SCNT extraembryonic tissue. As shown in Figure 3, histological analysis of SCNT extraembryonic tisssues showed clusters of terminal villi with apparent syncytial bridging and sprouting. Adhesion occurred between the maternal and fetal epithelium with the formation of microvilli resulting in an interdigitation of both tissues. The expression of antioxidant proteins GPX and catalase revealed extensive positive staining in the extraembryonic tissues of control fetus; however, SCNT-derived extraembryonic tissue exhibited significantly lower expression levels of GPX and catalase (Figure 3A and B).

### Western blot analysis in control and SCNT extraembryonic tissues

**Figure 4:**
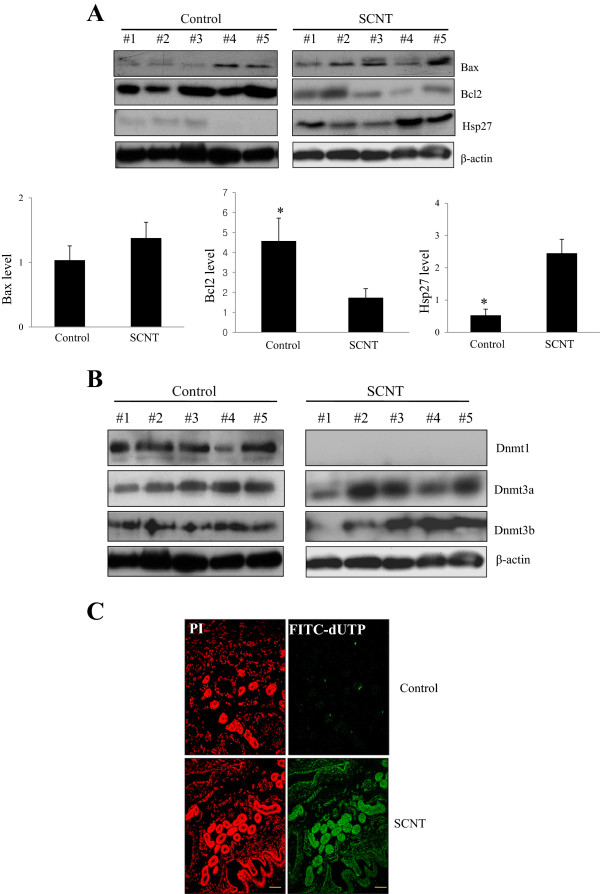
**Up-regulated apoptotic marker proteins in the SCNT extraembryonic tissue. (A)** The expression of Bax and Hsp27 were up-regulated and the Bcl2 expression was down-regulated in the SCNT extraembryonic tissue. Actin was used as a loading control. **(B)** Dnmt1 was highly expressed in the control extraembryonic tissue while the expression of Dnmt3a and 3b Dnmt3a and 3b were highly detectable in the SCNT extraembryonic tissue. **(C)** TUNEL assays in the control and SCNT extraembryonic tissue. Dewaxed paraffin sections from the control and SCNT extraembryonic tissue were subjected to the TUNEL assay. Fragmented DNAs were detected by a TUNEL assay using a fluorescence microscope with either propidium iodide (PI) (left) or FITC (FITC-dUTP) (right). In normal extraembryonic tissue, apoptotic cells were rarely identified while apoptotic cells significantly increased in SCNT extraembryonic tissue. Magnification: control and SCNT extraembryonic tissue (x200).

To further investigate whether the developmental defects in the extraembryonic tissue of SCNT fetus affect the expression of antioxidation-related proteins or apoptotic marker proteins, such as GPX, catalase, Bax, Bcl2, and Hsp27, we performed western blot analysis using control and SCNT extraembryonic tissue. The expression levels of catalase or GPX related to oxidative stress were decreased in SCNT extraembryonic tissues (Figure 3C). The expression of Hsp27 was up regulated more than two-fold in SCNT extraembryonic tissue compared with control tissue, and these data are consistent with 2-D analysis. The expression of Bax, an apoptotic marker protein, was up-regulated, and the expression of the anti-apoptotic Bcl-2 gene decreased in the SCNT extraembryonic tissue (Figure 4A). Next, we investigated the expression of DNA methyltransferases 1, 3a and 3b (Dnmt 1, 3a and 3b) in control and SCNT extraembryonic tissue. We found that that Dnmt1 was highly expressed in the extraembryonic tissue of control fetus, whereas the activation of the protein is restricted in the SCNT extraembryonic tissue (Figure 4B). In contrast, the expression levels of Dnmt3a and 3b were highly detectable in the SCNT extraembryonic tissue, while they were low in control fetus (Figure 4B). These results suggest that a relatively low level of Dnmt1 activity exists during the development of SCNT fetus.

### *TUNEL analysis of*control *and SCNT extraembryonic tissue*

To determine whether the high expression of apoptotic or antioxidant proteins is due to increased apoptosis in SCNT-derived extraembryonic tissue, the TUNEL assay was performed with the extraembryonic tissues from control and SCNT fetus. A small portion of the TUNEL-positive cells was observed in the extraembryonic tissue of control fetus (Figure 4C); however, marked apoptosis was observed in SCNT extraembryonic tissue (Figure 4C). The apoptotic cells in the SCNT extraembryonic tissue were trophoblast cells, which play an important role during pregnancy in the growth and development of the placenta. These results demonstrate that inefficient development of the extraembryonic tissue in SCNT fetus caused abnormal levels of apoptosis in SCNT-derived extraembryonic tissue.

## Discussion

In the present study, we investigated differences in the protein expression profiles between control and SCNT extraembryonic tissues at early pregnancy at gestational day 35. Our data showed that proteomics analysis of the extraembryonic tissue from SCNT fetus revealed that 33 proteins were differentially regulated compared to their expression in control fetus. We found that the gross differences in the protein expression patterns in the SCNT fetus, including genes involved in lipid binding proteins, translation elongation factors, ATP binding proteins, transcription factors, and metabolic regulation genes, were associated with placental abnormalities [15].

Pig extraembryonic tissue contains the trophoblast, a major component of the placenta that induces blastocyst attachment into the uterine wall, and regulates the development of both the fetal and maternal compartments of the placenta [16]. The result from the failure to develop a normal trophoblast was believed to be the primary cause of early fetus lethality and defects. In the present study, our results showed that the size and shape of the fetus and amniotic sac of control and SCNT fetus were similar. However, the extraembryonic tissue derived from SCNT fetus was abnormally small, and it did not develop to the same degree as control fetus. SCNT fetus in mice showed a large placenta size that was associated with hypertrophy of the basal layer, spongiotrophoblasts, giant trophoblasts, and glycogen cells [17]. These results obtained from pig SCNT fetus do not correspond to results obtained from mouse SCNT placentas, suggesting that these differences in placenta size are the result of different cell types of trophoblasts or implantation types in the uterus. These results strongly indicate that the extraembryonic tissue in SCNT fetus fails to develop normally, which causes subsequent abnormalities in implantation and the development of the placenta.

During the normal development of SCNT fetus, epigenetic modifications in the somatic nucleus occur during preimplantation development of the SCNT fetus, with the silencing of differentiation-associated genes and the activation of genes critical for fetus development. Recently, many studies demonstrate that global gene expression is similar in SCNT and control fetus; however, dysregulated gene expression is observed in the extraembryonic tisssues of SCNTs [18]. Most SCNT fetus display abnormalities in their DNA methylation patterns and abnormal expression of several imprinted and nonimprinted genes, suggesting a relationship between these aberrations and abnormalities in the development of SCNT fetus [19, 20]. Our data showed that the expression of Dnmt1 significantly decreased in SCNT extraembryonic tissue compared with extraembryonic tissue of a control fetus, whereas Dnmt3a and Dnmt3b were highly expressed in SCNT extraembryonic tissue. These results indicated that the expression of Dnmt1 is restricted in SCNT fetus and tightly controlled compared with control fetus, reflecting the occurrence in aberrant epigenetic reprogramming and aberrant protein expression in SCNT-derived extraembryonic tissue. Several studies demonstrated that cloned fetus frequently possess aberrant DNA methylation changes [21], and genome-wide aberrant demethylation was observed in cloned bovine fetus during preimplantation development [22]. Therefore, our data suggest that changes in DNA methylation may frequently occur in SCNT extraembryonic tissue, and it is possible that the cloned fetus are reprogrammed incompletely in their extraembryonic tissue and subsequently show abnormalities in protein expression.

Oxidative stress results from the endogenous or exogenous generation of reactive oxygen species (ROS), which damages macromolecules and causes apoptosis [23]. The decrease in the formation of ROS is derived by Superoxide dismutase, Glutathione Peroxidase (GPX), and catalase, which converts H_2_O_2_ to O_2_ and H_2_O [23]. In the present study, several antioxidant proteins were significantly down-regulated in the extraembryonic tissue of pig SCNT fetus. Our data showed that the catalase and GPX proteins were down-regulated in SCNT extraembryonic tissue via Western blotting analysis and immunohistochemistry. A recent study showed that increased oxygen demand leads to an increased rate of ROS production during embryonic development and oxidative stress, which contributes to ROS formation in the extraembryonic tissue of the placenta [24]. Moreover, SCNT extraembryonic tissue increased the expression of Hsp27, which induces apoptotic signaling by interacting with key components of the apoptosis pathway and activates the caspase signaling cascade [25]. The expression level of the antiapoptotic gene Bcl-2 was lower in SCNT extraembryonic tissue compared with control tissue, whereas the expression of the apoptotic marker gene Bax increased in the extraembryonic tissue of SCNT fetus. Taken together, these findings indicate that extraembryonic tissue from SCNT fetus induced apoptotic signaling, which was due to the upregulated expression of apoptosis-related proteins. Furthermore, our data showed that a small portion of the cells was TUNEL-positive in the extraembryonic tissue of control fetus, whereas a large portion of the cells was TUNEL-positive in SCNT extraembryonic tissue, indicating that apoptosis occurred in SCNT fetus. Together, our data suggested that oxidative stress or apoptosis-related proteins activate apoptotic signaling, and apoptosis occurred in the pig SCNT extraembryonic tissue. These results demonstrated that abnormal apoptosis in the extraembryonic tissue of SCNT fetus during early embryonic development leads to the early loss of cloned pig fetus.

## Conclusion

In the proteomic analysis using the extraembryonic tissue on the 35 day of pregnancy from the SCNT fetus, 33 proteins were identified as differentially regulated proteins. Among the up-regulated proteins, Hsp27 and Bax were found, which are closely related to the process of apoptosis. However, the expression levels of the catalase and GPX genes were down-regulated in SCNT fetus, suggesting that ROS accumulates in SCNT extraembryonic tissue and causes apoptosis. Therefore, we suggest that abnormal protein expression and apoptosis in the extraembryonic tissue of SCNT fetus is a primary reason for the high rates of embryonic death in pig SCNT fetus during early pregnancy.

## Methods

### Animal ethics

The treatment of the pigs used in this research abided the guidelines of the National Institute of Animal Science, Rural Development Administration, Suwon, South Korea, and was approved by the Institutional Animal Care and Use Committee (approval number: 2010–009, D-grade).

### Production of cloned embryos

Ovaries were obtained from prepubertal crossbred gilts at a local slaughterhouse and transported to the laboratory at 30-35°C. COCs that had several layers of cumulus cells were selected and washed three times in maturation medium. For the maturation culture, approximately 50–100 COCs were transferred to 500 μl of maturation medium (TCM-199, Gibco-BRL, Grand Island, NY, USA) covered with mineral oil in a four-well dish. Oocytes were matured at 38.5°C under 5% CO_2_ in air for 40 to 44 hours. Primary culture of fetal fibroblasts was established from the ear skin tissue of the crossbred pig by routine cell culture technology [8, 26]. The ear tissues were cut into small pieces and dispersed by exposure to 0.25% trypsin-0.02% EDTA (Gibco-BRL). Following digestion, the cells were centrifuged to removed debris and cultured in DMEM (Gibco-BRL) supplemented with 10% fetal bovine serum and 75 μg/ml of antibiotics. Fibroblast cells were cultured and passed (2 to 8 passages) and then used as donor cells for nuclear transfer. After maturation, cumulus cells were removed from oocytes by vortexing the COCs in PBS supplemented with 0.1% PVA and 0.1% hyaluronidase for 4 min. Oocytes were enucleated by aspirating the first polar body and metaphase-II (MII) plate in a small amount of surrounding cytoplasm with a glass pipette. All micromanipulation procedures were performed in TCM-199 supplemented with 3 mg/ml BSA and 5 μl/ml cytochalasin B. Enucleation was confirmed by staining the oocytes with 10 μg/ml Hoechst 33342 for 15–20 min at 39°C. After the enucleation, the oocytes were held in TCM-199 supplemented with 3 mg/ml BSA until injection of donor cells. Reconstructed oocytes were then placed between 0.2 mm diameter wire electrodes (1 mm apart) in a fusion chamber overlaid with 0.3 M mannitol solution supplemented with 0.1 mM MgSO_4_, 1.0 mM CaCl_2_, and 0.5 mM HEPES. For the fusion, two DC pulses of 1.2 kV/cm were applied for 30 μs using a BTX Electro Cell Manipulator 2001 (BTX, San Diego, CA, USA). After the fusion treatment, the reconstructed oocytes were cultured in TCM-199 supplemented with 3 mg/ml BSA for 1 hr, and fusion was determined. The basic culture medium used was PZM-3. SCNT embryos were cultured in PZM-3 for 1 or 2 days prior to embryo transfer.

### Extraembryonic tissues

Recipients were prepared as described previously [26]. The embryos produced by SCNT were cultured for 1 or 2 days. A total of 150 embryos that were morphologically normal at the 1–2 cell stages were selected for transfer. Embryos were then transferred into the oviducts of the recipient gilt approximately 48 h after human chorionic gonadotropin (hCG) injection. The pregnancy status of recipients was determined by ultrasound between days 30 and 33 after embryo transfer. The extraembryonic tissues including cholioallantois, umbilical cord, and placenta were obtained from the uterus of pregnant gilts at day 35 of gestation after transfer of SCNT embryos or natural mating (normal embryos). The size and shape of extraembryonic tisssues were observed in the microscopic analysis. Three extraembryonic tissues surrounding each fetus which were recovered from three recipients were subjected to subsequent experiments.

### 2-D gel analysis and protein identification

2-D and spot analysis were performed as previously described [8]. From control and SCNT extraembryonic tissues were solubilized in lysis buffer containing 7 Murea, 2 M thiourea, 4% w/v CHAPS, 40 mM DTT, and 0.5% Pharmalyte at pH 4–7. Insoluble material was removed by centrifugation. Total proteins (1000 μg) for analytical runs were transferred into IPG strips (17 cm, pH 4–7, BioRad) holder channels (BioRad, CA, USA). 2-D separated protein mixtures by IEF in the first dimention and SDS-PAGE in the second dimention. The resulting gels provided a high-resolution separation of a complex mixture of proteins. The gels were stained with silver staining to visualize proteins. Silver staining kit (Amersham Biosciences) was used for 2-D gels. Three or four independent gels were performed in triplicate. The gels were fixed in 40% ethanol and 10% acetic acid for 30 min and sensitized in ethanol glutardialdehyde (25%, w/v), sodium thiosulfate (5%, w/v), and sodium acetate for 30 min followed by three washes with water for 15 min each. Then the gels were immersed in silver nitrate (2.5%, w/v) and formaldehyde (37%, w/v) for 20 min, developed with sodium carbonate and formaldehyde (37%, w/v) for 5 min, and stopped in EDTA-Na2-2H2O. Gels were scanned and Image analysis was performed using Progenesis Samespots software (Nonlinear Dynamics, Newcastle, UK). Using this software, the differentially expressed spots were identified by automatic matching of the detected protein spots. Those spots differing significantly (p < 0.05, ANOVA test) in their intensities with a fold-change ≥2 were used for further analysis. Target spots, identified using PDQuest software (BioRad), were excised from the gel, destained, and subjected to in-gel digestion with bovine trypsin (Promega, USA). Briefly, trypsin digestion reactions were terminated with TFA (final concentration 10%). Peptides were concentrated and desalted using ZipTips (m-c18, Millipore, Etten-Leur, The Netherlands) and eluted directly onto the MALDI target in 1 mL of a saturated solution of CHCA in 50% ACN. Peptides were analyzed using a Voyager DE-STR MALDI-TOF mass spectrometer (Applied Biosystems, Framingham, MA, USA) in reflection mode, at a 20 kV accelerating voltage.

### Immunohistochemical analysis

Tissues were fixed in 4% w/v paraformaldehyde in 0.01 M PBS (pH 7.4), washed in PBS, dehydrated in a series of ethanol washes (70, 90, and 100%) and embedded in paraffin. Sections were rehydrated (xylene for 5 min; 100, 95, 70% ethanol, 2 min each) and washed in distilled water prior to TUNEL staining. For TUNEL staining, sections were incubated for 15 min with proteinase K (20 mg/mL) at room temperature, washed with PBS, and then incubated in 2% H_2_O_2_ for 5 min to block the endogenous peroxidase activity. Sections were rewashed three times with PBS and incubated for 60 min at 37C in a moist chamber with TUNEL mixtures (0.3 U/mL calf thymus terminal deoxynucleotidyl transferase, 7 pmol/mL biotin dUTP, and 1 mM cobalt chloride in 16 reaction buffer in distilled water). After washing in four PBS baths of 5 min each at room temperature, the sections were saturated in 2% BSA for 10 min at RT. Sections were treated for 30 min at 37°C in a moist chamber with a 1:20 dilution of ExtraAvidin peroxidase antibody. After three PBS washes, detection was performed with DAB (1.24 mg DAB, 25 mL 3% NiCl_2_, 152 mL 1 M Tris–HCl (pH 7.5) in 2 mL distilled water). Slides were mounted in crystal mount (Biomeda, Foster City, CA). Immunohistochemical staining was performed with an ABC Kit (Oncogene Science, MA, USA), according to the manuscript’s instruction. Sections were washed in water and mounted with a cover slip.

### Western blot analysis

To further examine the differentially expressed genes between the control and SCNT extraembryonic tissues, we performed Western blot analysis using control and SCNT extraembryonic tissue. For Western blot analysis, 50 μg of soluble protein were loaded onto a 10% SDS/polyacrylamide gel and separated by electrophoresis, followed by electrotransfer to a PVDF membrane (Immobilon-P, Millipore) using a semidry blotting apparatus and a low ionic discontinuous buffer system. The membrane was incubated in TBST containing 5% skim milk and 0.05% Tween-20 and hybridized with primary antibodies. GPX, Catalase, Bax, Bcl2, and Hsp27 antibody were obtained from Cell Signaling Technology (MA, USA), Dnmt1, 3a, and 3b were from Santa Cruz Technology (Santa Cruze, CA, USA), and the monoclonal β-actin antibody was from Millipore (Darmstadt, Germany). HRP-labeled mouse anti-rabbit IgG were from Jackson ImmunoResearch. The Chemiluminescence kit was from Pierce (Rockford, IL). After incubation with horseradish-peroxidase-conjugated secondary antibody at room temperature, immunoreactive proteins were detected using a chemiluminescent ECL assay kit (Amersham Pharmacia, UK) according to the manufacturer’s instructions.

### Statistical analysis

Data are expressed as means ± SD. Significant difference among the treatment means were determined using ANOVA and Ducan’s multiple range test at p <0.05.
